# Proteomic analysis allows for early detection of potential markers of metabolic impairment in very young obese children

**DOI:** 10.1186/1687-9856-2014-9

**Published:** 2014-06-10

**Authors:** Gabriel Á Martos-Moreno, Lucila Sackmann-Sala, Vicente Barrios, Darlene E Berrymann, Shigeru Okada, Jesús Argente, John J Kopchick

**Affiliations:** 1 Edison Biotechnology Institute, Ohio University, Konneker Research Laboratories, 1 Water Tower Drive, The Ridges, 45701 Athens, Ohio, USA; 2 Hospital Infantil Universitario Niño Jesús, Department of Endocrinology, Instituto de Investigación la Princesa, Universidad Autónoma de Madrid, Department of Pediatrics, Av. Menéndez Pelayo, 65, E-28009 Madrid, Spain; 3 CIBER Fisiopatología Obesidad y Nutrición (CIBERobn), Instituto de Salud Carlos III, Madrid, Spain; 4 Department of Biological Sciences, College of Arts and Sciences, Ohio University, Athens, Ohio, USA; 5 Molecular and Cellular Biology Program, Ohio University, Athens, Ohio, USA; 6 Department of Biomedical Sciences, College of Osteopathic Medicine, Ohio University, Athens, Ohio, USA

**Keywords:** Proteomic, Two dimension electrophoresis, Childhood obesity, Insulin resistance

## Abstract

**Background:**

Early diagnosis of initial metabolic derangements in young obese children could influence their management; however, this impairment is frequently not overt, but subtle and undetectable by routinely used clinical assays. Our aim was to evaluate the ability of serum proteomic analysis to detect these incipient metabolic alterations in comparison to standard clinical methods and to identify new candidate biomarkers.

**Methods:**

A cross-sectional study of fasting serum samples from twenty-two prepubertal, Caucasian obese (**OB**; 9.22 ± 1.93 years; 3.43 ± 1.08 BMI-SDS) and twenty-one lean controls (**C**; 8.50 ± 1.98 years; -0.48 ± 0.81 BMI-SDS) and a prospective study of fasting serum samples from twenty prepubertal, Caucasian obese children (11 insulin resistant [**IR**]) before (4.77 ± 1.30 BMI-SDS) and after weight reduction (2.57 ± 1.29 BMI-SDS) by conservative treatment in a reference hospital (**Pros-OB**) was performed. Proteomic analysis (two-dimension-eletrophoresis + mass spectrometry analysis) of serum and comparative evaluation of the sensitivity of routinely used assays in the clinics to detect the observed differences in protein expression level, as well as their relationship with anthropometric features, insulin resistance indexes, lipid profile and adipokine levels were carried out.

**Results:**

Study of the intensity data from proteomic analysis showed a decrease of several isoforms of apolipoprotein-A1, apo-J/clusterin, vitamin D binding protein, transthyretin in **OB***vs*. **C**, with some changes in these proteins being enhanced by **IR** and partially reversed after weight loss. Expression of low molecular weight isoforms of haptoglobin was increased in **OB**, enhanced in **IR** and again decreased after weight loss, being positively correlated with serum interleukin-6 and NAMPT/visfatin levels. After statistical correction for multiple comparisons, significance remained for a single isoform of low MW haptoglobin (OB vs. C and IR vs. non-IR) and Apo A1 (IR vs. non-IR). Assays routinely used in the clinical setting (ELISA/kinetic nephelometry), only partially confirmed the changes observed by proteomic analysis (ApoA1 and haptoglobin).

**Conclusion:**

Proteomic analysis can allow for the identification of potential new candidate biomarkers as a complement to routinely used assays to detect initial changes in serum markers of inflammation and lipid metabolism impairment in young obese children.

## Background

Early-onset obesity is a definite risk factor for adult obesity, its associated comorbidities and reduced life expectancy [1,2]. Most of the metabolic comorbidities overtly observed in obese adults are usually subtle or even undetectable in most young (prepubertal) obese children when the currently available biomarkers, including the growing number of adipokines, are used. Adipokines play a preeminent role in carbohydrate metabolism, the derangement of which is the most important metabolic comorbidity of obesity, which can ultimately lead to type 2 diabetes mellitus ([T2DM] [3,4]). Impaired adipokine synthesis and secretion, which can be detected even in young obese children, contributes to the generation of insulin resistance (IR) in a dual fashion. Obesity induces changes in serum levels of insulin sensitizing adipokines [4-7] and also generates a generalized proinflammatory environment by increasing the circulating levels of resistin, interleukin 6 (IL-6) and tumoral necrosis factor alpha (TNF-α) [6,8]. However, these changes in circulating adipokines related with carbohydrate metabolism have not been shown to be of value for the detection of early stages of homeostatic derangement in young obese children. Hence, there is a lack of sensitive markers for the initial stages of carbohydrate metabolism impairment in childhood obesity. A similar situation is observed with regard to the obesity related impairment of lipid metabolism, mainly represented by the decrease in high density lipoprotein (HDL) levels in this age range and definitely influenced by the existence of IR.

Consequently, analysis of the serum proteome of young children in different weight-related conditions (normal, excess and later reduction), with or without IR, could be useful for the detection of new more sensitive diagnostic biomarkers. An advantageous tool for the study of the circulating proteome in serum under different conditions is the use of two dimension electrophoresis (2DE). This technique allows for the isolation and identification of proteins in a given sample and has previously been applied to human serum samples for different purposes, with a limited amount (below 0.5 ml) of serum needed to identify up to 300 protein spots [9-12]. Therefore, the aims of this study were: 1) To investigate the influence of obesity and weight loss on the serum proteomic profile of young prepubertal obese children. 2) To evaluate the differences in the serum proteome between young obese children according to the presence or lack of estimated IR and 3) To identify new candidate biomarkers of early metabolic impairment in young obese children.

## Subjects and methods

### Subjects

Three groups of children were enrolled for this study: obese patients (**OB**), controls (**C**) and a second cohort of prospectively followed obese patients (**Pros-OB**).

#### ***Obese patients (OB)***

This group was made up of twenty-two prepubertal (Tanner stage I) obese (BMI > +2 SDS according to Spanish standards [13]) Caucasian children (13 males / 9 females). Their mean age at recruitment was 9.22 ± 1.93 years (range 5.53-12.89) and their mean BMI 3.43 ± 1.08 SDS. Samples from these patients were obtained exclusively at the time of enrollment in the study.

#### ***Control group (C)***

This group was made up of twenty-one prepubertal (Tanner stage I) lean (BMI between -1.5 and + 1.5 SDS according to Spanish standards [13]) Caucasian children (16 boys/ 4 girls). Their mean age at recruitment was 8.50 ± 1.98 years (range 5.64-11.95) and their mean BMI -0.48 ± 0.81 SDS. These children were referred to our department but found to be healthy, with no pathological auxological, clinical or analytical findings. Samples from controls were obtained exclusively at the time of enrollment in the study.

#### ***Prospectively followed obese patients (Pros-OB)***

A subgroup of a larger cohort of obese patients that were prospectively followed for 18 months in an intensive program of behavioural modification [6] was used in these studies. It was made up of twenty children, all of whom had achieved extensive BMI reduction (over 2 SDS regarding their BMI at enrollment) during the first 12 months of follow-up. All of them were prepubertal (Tanner stage I), obese (BMI > +2 SDS according to Spanish [13]) and Caucasian (16 boys/ 4 girls). Their mean age at recruitment was 8.64 ± 1.56 years (range 5.44-11.70) and serum samples were obtained at two different time-points: at diagnosis (**D**, mean BMI 4.77 ± 1.30 SDS) and after reducing their BMI by over 2 SDS (weight loss [**WL**], mean BMI 2.57 ± 1.29 SDS). These patients underwent an oral glucose tolerance test at D (OGTT, 1.75g/kg, maximum 75g) to estimate their degree of IR. Body composition analyses were performed by DXA (Hologic QDR4500W) at time-points D and WL, confirming that the observed weight loss was due exclusively to body fat mass reduction, without bone density or lean mass impairment [6]. Blood samples were obtained at both time-points (D and WL).

All samples were obtained after overnight fasting. Those samples from children in groups OB and C were centrifuged and serum stored at –80°C until preparation for proteomic analysis. The samples from the patients in the Pros-OB, both at D and at WL, underwent a lyophylization process for better preservation after collection and were reconstituted immediately before preparation for proteomic analysis.

All patients and controls and their parents or guardians gave informed consent as required by the local ethics committee, which had previously approved the study. The study was also approved by the Ohio University Institutional Review Board.

### Biochemical measurements

Serum lipid profile (triglycerides, total cholesterol, HDL, LDL and VLDL), glucose, insulin, total (T) and high molecular weight (HMW) adiponectin, resistin, IL-6, TNF-α, NAMPT/visfatin and vaspin levels were measured in separated aliquots of each sample by using commercial assays as previously reported [6,7]. The findings in proteomic analysis were contrasted with validated commercial assays by ELISA [Apolipoprotein-A1 (Apo-A1; Assaypro^(R)^), haptoglobin (Assaypro^(R)^), apo-J/clusterin (BioVendor^(R)^), alpha-1B-acid glycoprotein (α-1-BAGP; Assaypro^(R)^), alpha-1-antitrypsin (α1-AT; Assaypro^(R)^); vitamin D binding protein (vD-BP; R&D Systems^(R)^); retinol binding protein 4 (RBP4; R&D Systems^(R)^)] or kinetic nephelometry [transthyretin (Immage. Beckman^(R)^].

HOMA index was calculated as glucose [mmol/l] × insulin [μUI/ml]/22.5 and S_A_ as HMW-(μg/ml)/(T)-adiponectin(μg/ml). IR was estimated in patients in the **Pros-OB** at **D** according to the patient’s insulin secretion rate in the OGTT and classified at baseline as insulin resistant (**IR**, n = 11) if they met any of the following criteria: basal insulin > 15 μU/ml, peak insulin > 150 μU/ml or insulin at 120 minutes > 75 μU/ml or as not IR (**non-IR**, n = 9) if they did not [14].

### Sample preparation for proteomic analysis

The serum samples were thawed or reconstituted (according to their preservation process). Fifty milliliters of each selected serum sample were albumin and Ig-G-depleted using an albumin and IgG Depletion kit (ProteoPrep^®^ Blue Albumin Depletion Kit, Sigma-Aldrich, St. Louis, MO) and diluted in sample buffer containing 7 M urea, 2 M thiourea, 1% w/v SB 3–10, 3% w/v CHAPS, 0.25% v/v Bio-Lyte 3/10 ampholytes (Bio-Rad Laboratories Inc., Hercules, CA), and 1.5% v/v protease inhibitor cocktail (Sigma, St. Louis, MO). Disulfide bonds were reduced by addition of tributylphosphine and sulfhydryl groups were alkylated with iodoacetamide.

### Two-dimensional gel electrophoresis (2DE) + mass spectrometry (MS)

All of the 2DE, image processing and analysis, mass spectrometry (MS) and tandem MS (MS-MS) analysis procedures (MALDI-TOF) have been detailed previously for human samples [9-12,15]. Identities obtained after MS and MS-MS analysis were verified using the MS and MS/MS data obtained to search online databases with the software Mascot (http://www.matrixscience.com) [9-12,15].

### Sets of comparison

Due to the different sample processing and preservation procedures undergone by the samples from the patients in the Pros-OB group (lyophylization), regarding the OB and C groups (exclusively freezing), the obese patients from these groups were not pooled for analysis at baseline. The following sets of comparison were established in order to achieve the proposed objectives avoiding any possible bias derived from differences in the process of sample preservation:

1) Influence of obesity on serum proteomic profile: OB (n = 22) *vs.* C (n = 21).

2) Differences in the serum proteome due to insulin resistance: Pros-OB group at baseline (B): IR (n = 11) *vs.* non-IR (n = 9).

3) Effect of extensive weight reduction on serum proteomic profile: Pros-OB: Paired comparison between time-points D and WL (n = 20 at each time-point).

### Statistical analysis

All 2DE intensity data were log-transformed. Variables fitting a normal distribution (p > 0.05 in a Shapiro–Wilk test) were compared between pairs of groups using a Student-t test for independent samples (OB *vs.* C; IR *vs.* non-IR) or a paired t-test for repeated samples (D *vs.* WL). The remaining variables were analyzed using the nonparametric Mann & Whitney U test (OB *vs.* C; IR *vs.* non-IR) or Wilcoxon test (D *vs*. WL). Correlation analysis was performed by using Pearson’s r or Spearman’s rho for parametric and non parametric variables, respectively. A value of *p* < 0.05 was chosen as the level of significance. These tests were performed using the software Statistical Package for Social Sciences (SPSS v. 14.0. MapInfo Corporation, Troy, NY, USA). Due to the high number of variables considered for every comparison set, statistical correction for multiple comparisons was performed by using the false discovery rate (FDR) test after initial comparisons (PRISM v. 6.0. GraphPad Software, Inc., La Jolla, CA, USA).

## Results

### Influence of obesity on the serum proteomic profile

**Figure 1:**
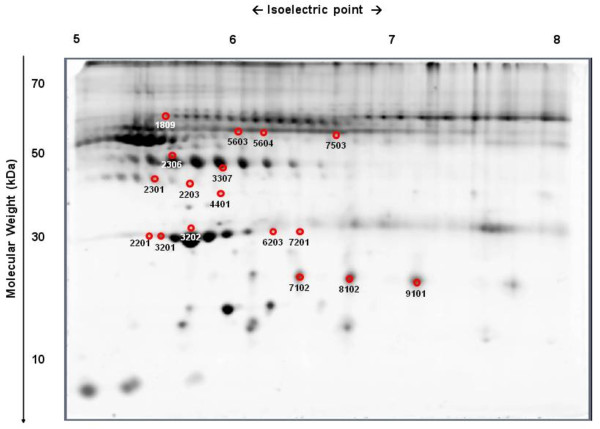
**Proteomic profile comparison of OB vs. C.** Protein identification of spots displaying differences (p < 0.05) between obese (OB) and control (C) children before FDR analysis. For spot identification, please see Table 1.

The study of the serum proteomic profile in OB (n = 22) and C (n = 21) allowed for the analysis of 231 protein spots. The comparison of the protein spot intensities revealed that 17 of them showed differences between groups (p < 0.05). Among these, 6 proteins were over-expressed in OB compared to C, whereas the expression of the remaining 11 protein spots was decreased in OB (Table 1, Figure 1).

**Table 1:** Protein spot displaying intensity differences (p < 0.05) between obese children (OB) and controls (C) before FDR analysis

***Spot***	***Spot intensity in OB***	***MS***	***MS***	***MS***	***MS -MS***	***MS-MS***	***MS-MS***	***Identification***
	***Significance level***	***Score/Cut-off***	***Matched fragments***	***Sequence coverage (%)***	***Score/Cut-off***	***Matched (Significant) fragments***	***Sequence coverage (%)***	

1809	Upregulated, p < 0.05	68/66	15	26	45/40	2 (1)	6	*Alpha 1B acid glycoprotein*
2201	Downregulated, p < 0.01	115/66	12	50	98/41	9 (6)	40	*Apolipoprotein A1*
2203	Downregulated, p < 0.05	44/66	3	--	102/41	3(3)	6	*Clusterin (Apolipoprotein J)*
2301	Downregulated, p < 0.05	56/66	7	--	95/41	5(4)	23	*Clusterin (Apolipoprotein J)*
2306	Upregulated, p < 0.05	75/66	7	30	71/41	6(2)	17	*Haptoglobin*
3201	Downregulated, p < 0.05	137/66	16	59	81/41	8(4)	33	*Apolipoprotein A1*
3202	Downregulated, p < 0.01	71/66	8	34	81/41	6(4)	25	*Apolipoprotein A1*
3307	Upregulated, p < 0.05	91/66	7	36	61/58	3(1)	6	*Haptoglobin*
4401	Downregulated, p < 0.05	68/66	6	83	95/59	2(2)	*28*	*Transthyretin chain A*
5603	Downregulated, p < 0.05	71/66	6	11	79/59	3(1)	*6*	*Haptoglobin*
5604	Downregulated, p < 0.01	82/66	11	49	84/58	*2(1)*	*4*	*Haptoglobin*
6203	Downregulated, p < 0.05	127/66	17	65	76/58	6(3)	*29*	*Apolipoprotein A1*
7102	Upregulated, p < 0.01	55/66	8	--	64/56	2(1)	*13*	*Haptoglobin (alpha2)*
7201	Downregulated, p < 0.01	106/66	16	66	76/59	3(2)	*13*	*Apolipoprotein A1*
7503	Downregulated, p < 0.05	53/66	5	--	64/58	2(2)	*7*	*Vitamin D binding protein*
**8102**	**Upregulated, p < 0.01**	**68/66**	**5**	**20**	**70/57**	**3(1)**	***8***	***Haptoglobin (alpha2)***
9101	Upregulated, p < 0.05	49/66	3	--	105/55	1(1)	*4*	*Haptoglobin (alpha2)*

Protein spots displaying significant intensity differences between obese children (**OB**) and controls (**C**) prior to the false discovery rate (FDR) analysis. The spot in bold (8102) remained significantly different after FDR analysis. The second column shows the direction of the change observed in **OB***vs.***C**. *Abbreviations:MS* Mass spectrometry; *MS-MS* Tandem mass spectrometry.

Among the observed changes, the down-regulation of several isoforms of Apo A1 (#2201, #3201, #3202; #6203; #7201), clusterin/Apo J (#2203; #2301), vD-BP (#7503) and transthyretin (#4401) was remarkable. OB patients showed a decreased expression of the highest molecular weight (MW) isoforms of haptoglobin (#5603, #5604), whereas those isoforms with a medium (#2306; #3307) or low MW (#7102; #8102; #9101) were up-regulated, as was α-1-BAGP (#1809).

After FDR analysis, only differences between groups in spot #8102 (low MW haptoglobin) remained significant (p < 0.001).

When analyzed by ELISA, lower serum levels of Apo-A1 (120.7 ± 56.9 *vs.* 236.3 ± 91.7 mg/dl, p < 0.001) in OB were observed, as was HDL cholesterol (46.22 ± 10.64 *vs.* 62.87 ± 12.53 mg/dl, p < 0.001). Similarly, ELISA was successful in discriminating the increase in α-1-BAGP (179.3 ± 78.2 *vs.* 125.8 ± 49.1; p < 0.01) and total haptoglobin (162.5 ± 103.5 *vs.* 85.84 ± 60.63 mg/dl) in OB *vs.* C.

In contrast, ELISA assays did not detect differences between OB and C in serum clusterin/Apo J (61.07 ± 9.75 *vs.* 57.29 ± 12.81 mcg/ml) or vD-BP levels (223.1 ± 62.5 *vs.* 236.2 ± 71.2 mcg/ml). No differences between groups were found after transthyretin quantification by kinetic nephelometry (22.72 ± 6.23 vs. 22.71 ± 4.91 mcg/ml), nor in its substrate (RBP4) serum levels by ELISA (750.3 ± 206.8 vs. 794.9 ± 173.2 mcg/ml).

To explore the eventual effect of excess BMI on the expression of these protein spots, correlation studies were performed in the whole study group (22 OB + 21 C: n = 43) between the relative intensity of spots showing differences between groups prior to the FDR analysis and the BMI-SDS of the patients. As expected, significant negative correlations were found between BMI-SDS and several isoforms of Apo-A1, clusterin/Apo J , vD-BP and haptoglobin isoforms with high MW, whereas positive correlation coefficients were observed exclusively with those haptoglobin isoforms with a lower MW, including spot #8102 (Table 2).

**Table 2:** Correlation between BMI-SDS and protein spot intensity

***Protein***	***Correlation***	***Protein***	***Correlation***
#2201 (ApoA1)	r = -0.41; p < 0.01	#5604 (Haptoglobin)	r = -0.42; p < 0.05
#2203 (Clusterin)	r = -0.38; p < 0.05	#6203 (ApoA1)	r = -0.36; p < 0.05
#2301 (Clusterin)	r = -0.32; p < 0.05	#7102 (HP)	r = +0.36; p < 0.05
#3201 (ApoA1)	r = -0.32; p < 0.05	#7201 (ApoA1)	r = -0.35; p < 0.05
#3202 (ApoA1)	r = -0.43; p < 0.05	#8102 (HP)	r = +0.44; p < 0.01
#3307 (HP)	r = +0.32; p < 0.05	#9101 (HP)	r = +0.44; p < 0.01
#5603 (HP)	r = -0.35; p < 0.05		

Correlation coefficients (Spearman’s rho) and significance levels between patient’s standardized BMI (SDS) and protein spot intensity in the whole cohort of 43 prepubertal children studied, made up of 22 obese (**OB**) and 21 controls (**C**). *Abreviations:ApoA1* Apoprotein A1, *HP* Haptoglobin.

### Effect of insulin resistance on serum proteomic profile

**Figure 2:**
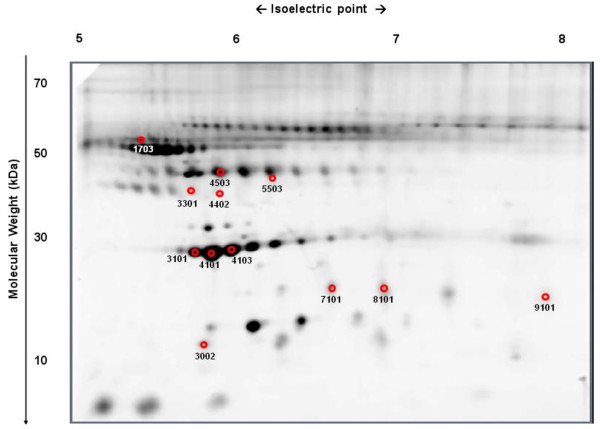
**Proteomic profile comparison of IR vs. non-IR.** Protein identification of spots displaying differences (p < 0.05) between insulin resistant (IR) and non insulin resistant (non-IR) obese children before FDR analysis. For the spot identification, please see Table 3.

Comparison of the serum proteomic profile between IR (n = 11) and non-IR (n = 9) obese children (the Pros-OB group at diagnosis) allowed for the analysis of 237 protein spots, with 12 of them showing differences between groups (p < 0.05). Among these, 5 proteins were over-expressed in IR compared to non-IR obese children, whereas the expression of the remaining 7 protein spots was decreased in IR (Table 3, Figure 2).

**Table 3:** Protein spot displaying intensity differences (p < 0.05) between insulin resistant (IR) and non-IR obese children before FDR analysis

***Spot***	***Spot intensity in IR,***	***MS***	***MS***	***MS***	***MS -MS***	***MS-MS***	***MS-MS***	***Identification***
	***Significance level***	***Score/Cut-off***	***Matched fragments***	***Sequence coverage (%)***	***Score/Cut-off***	***Matched fragments***	***Sequence coverage (%)***	
1703	Upregulated, p < 0.05	111/66	15	54	100/40	6(4)	26	*Alpha-1-Antitrypsin*
3002	Upregulated, p < 0.05	48/66	4	--	52/38	1(1)	4	*Haptoglobin*
3101	Downregulated, p < 0.05	160/66	20	69	116/40	11(8)	43	*Apolipoprotein A1*
3301	Downregulated, p < 0.05	85/66	7	22	79/41	5(4)	23	*Clusterin (Apo J)*
**4101**	**Downregulated, p < 0.01**	**165/66**	**23**	**73**	**150/49**	**11(5)**	**43**	***Apolipoprotein A1***
4103	Downregulated, p < 0.05	161/66	22	73	171/59	11(4)	43	*Apolipoprotein A1*
4402	Downregulated, p < 0.05	49/66	5	--	97/58	1(1)	4	*Clusterin (Apo J)*
4503	Downregulated, p < 0.05	93/66	13	33	72/59	6(2)	15	*Haptoglobin*
5503	Downregulated, p < 0.05	56/66	6	--	81/59	2(0)	*4*	*Haptoglobin*
7101	Upregulated, p < 0.05	74/66	6	20	99/56	2(1)	*7*	*Haptoglobin*
**8101**	**Upregulated, p < 0.05**	**55/66**	**5**	**--**	**73/56**	**2(1)**	***13***	***Haptoglobin***
9101	Upregulated, p < 0.05	49/66	3	--	105/55	1(1)	*4*	*Haptoglobin*

The second column shows the direction of the change observed in IR vs. non-IR. *Abbreviations:MS* Mass spectrometry, *MS-MS* Tandem mass spectrometry.

Spots in bold remained significant after FDR analysis.

Among the observed changes, it was remarkable that the presence of IR in obese children induced a further down-regulation of isoforms of Apo-A1 (#3101; #4101; #4103) and clusterin/Apo J (#3301; #4402). In contrast, no significant differences between the IR and non-IR groups were found in serum levels of clusterin/Apo J (54.67 ± 14.70 vs. 56.87 ± 14.80 mg/dl), Apo-A1 (235.4 ± 74.4 vs. 250.5 ± 79.4 mg/dl) or HDL (45.79 ± 8.82 vs. 45.89 ± 10.40 mg/dl) using standard immuno and enzymatic assays.

IR obese children also showed an increased expression of the 4 low MW isoforms of haptoglobin (#3002; #7101; #8101; #9101) and one of α1-AT (#1703), whereas the haptoglobin isoforms with the highest MWs (#4503; #5503) were down-regulated. When analyzed by ELISA, no significant differences between the IR and non-IR groups were found in serum levels of haptoglobin (202.7 ± 109.6 vs. 149.5 ± 49.5) or α1-AT (124.6 ± 60.1 vs. 115.2 ± 70.8).

After FDR analysis, differences between groups in spots #4101 (Apo A1) and #8101 (low MW haptoglobin) remained significant (both p < 0.0001).

To explore the relationship between the level of expression of these proteins and insulin resistance, the correlation between the relative intensity of protein spots showing differences between groups and the HOMA index of each patient in the entire study group was determined. As expected, significant negative correlations were found between HOMA and several isoforms of Apo-A1 (including spot #4101; rho -0.40 to -0.48; p < 0.05) and clusterin/Apo J (rho -0.45; p < 0.05), whereas positive correlation coefficients were observed with low MW isoforms of haptoglobin (including spot #8101; r = +0.55 to +0.61; p < 0.01).

Similar correlation studies were performed between these protein spot intensities at diagnosis and circulating levels of adipokines and the HMW to T adiponectin ratio (S_A_ index). No significant correlations were found for HMW adiponectin, T- adiponectin or S_A_, or for resistin, TNF-alpha or vaspin. In contrast, a significant positive correlation was found between serum IL-6 levels and the intensity of protein spots #8101 (r = +0.53; p < 0.05) and #9101 (r = +0.70; p < 0.01), both corresponding to low MW isoforms of haptoglobin. Serum visfatin levels were negatively correlated with the intensity of spot #3301 (clusterin/Apo J); r = -0.54; p < 0.05.

### Effect of extensive weight reduction on the serum proteomic profile

**Figure 3:**
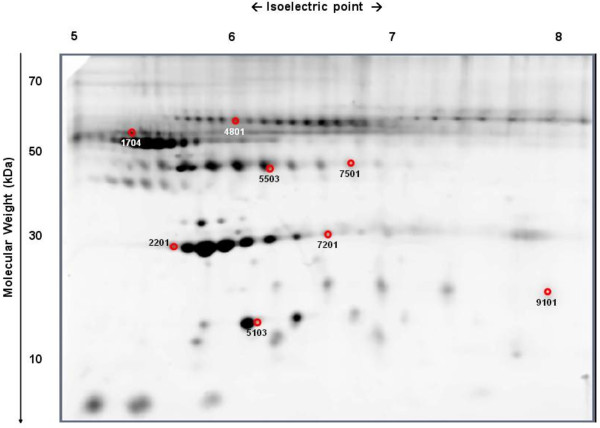
**Proteomic profile comparison in Pros-OB: D *vs. *WL.** Protein identification of spots displaying intensity differences (p < 0.05) in obese children (Pros-OB) between baseline (D) and after extensive BMI reduction (WL). For the spot identification, please see Table 4.

The paired comparison of the serum proteomic profiles of 20 obese children (the Pros-OB group), at diagnosis (D) and after reducing their BMI in over 2 SDS (WL) (n = 20 at each time-point) allowed for the analysis of 237 protein spots. Comparison of protein spot intensities revealed that 8 of them showed significant differences with 5 proteins increasing after weight loss and the remaining 3 protein spots decreasing as a consequence of BMI reduction (Table 4, Figure 3).

**Table 4:** Protein spot displaying intensity differences (p < 0.05) in pros-OB group after 2 SDS-BMI reduction

***Spot***	***Spot intensity after weight loss***	***MS***	***MS***	***MS***	***MS-MS***	***MS-MS***	***MS-MS***	***Identification***
	***Significance level***	***Score/Cut.off***	***Matched fragments***	***Sequence coverage***	***Score/Cut-off***	***Matched fragments***	***Sequence coverage***	
1704	Downregulated, p < 0.05	126/66	18	64	114/40	10(5)	37	***Alpha-1-Antitrypsin***
2201	Upregulated, p < 0.01	44/66	3	--	91/41	2(2)	12	***Apolipoprotein A1***
4801	Downregulated, p < 0.01	90/66	8	25	88/59	4(1)	12	***Alpha-1B-glycoprotein***
5103	Upregulated, p < 0.05	117/66	8	87	159/57	5(2)	79	***Transthyretin***
5503	Upregulated, p < 0.05	56/66	6	--	81/59	2(0)	4	***Haptoglobin***
7201	Upregulated, p < 0.01	106/66	16	66	76/59	3(2)	13	***Apolipoprotein A1***
7501	Upregulated, p < 0.05	55/66	4	--	86/59	2(2)	4	***Haptoglobin***
9101	Downregulated, p < 0.05	49/66	3	--	105/55	1(1)	4	***Haptoglobin***

The second column shows the direction of the change observed after weight loss. *Abbreviations:MS* Mass spectrometry, *MS-MS* Tandem mass spectrometry.

Among the observed changes it was remarkable that weight loss in obese children ameliorated the obesity induced decrease in Apo-A1 by increasing the expression of two of its isoforms (#2201; #7201), as well as increasing transthyretin (#5103). No significant differences in HDL levels (45.83 ± 9.30 vs. 49.42 ± 11.78 mg/dl) were found after weight loss when using standard assays, but Apo-A1 ELISA confirmed the increase in ApoA1 levels after weight reduction (242.6 ± 75.0 vs. 342.7 ± 83.9, p < 0.01). Similarly, no influence of weight loss on transthyretin (19.01 ± 3.95 vs. 19.66 ± 3.56 mg/dl) or RBP4 serum levels (572.5 ± 150.1 vs. 574.1 ± 127.6 mg/dl) were observed by kinetic nephelometry and ELISA, respectively.

Weight loss also decreased the expression of one isoform of low MW haptoglobin (#9101), whereas two haptoglobin isoforms with higher MW (#5503; #7501) were up-regulated after BMI reduction. In contrast, α1-AT (#1704) and α-1-BAGP (#4801) showed a decrease in their expression after weight loss. Standard assays were unable to detect significant changes after weight loss in serum levels of haptoglobin (177.5 ± 88.1 vs. 152.8 ± 55.6 mg/dl), α1-AT (120.2 ± 63.7 vs. 96.3 ± 71.1 mg/dl) or α-1-BAGP (156.0 ± 53.9 vs. 169.7 ± 43.8 mg/dl).

## Discussion

In this study we have shown that obesity can result in changes in the circulating serum proteome even at young ages. The intensity of these changes depends upon the degree of BMI excess in some cases and could be, at least partially, ameliorated through weight loss. We have also demonstrated how insulin resistance, the first step in obesity associated impairment of carbohydrate metabolism, enhances these changes in the degree of expression of specific isoforms of proteins related to inflammation (haptoglobin) and metabolism (ApoA1), some of them correlating with serum levels of adipokines, such as IL-6 and NAMPT/visfatin, with a known role in IR. Finally, we have demonstrated that serum proteomic analysis can complement the clinically used standard methodology in detecting these initial changes in the concentration of isoforms of circulating proteins associated with obesity related metabolic impairment and can identify new candidate biomarkers of early metabolic impairment.

One of the proteins significantly found to be affected in obesity associated IR is Apo-A1. There are two major classes of Apo-A; Apo-A1 and Apo-A2, with the former constituting the main component of HDL molecules [16]. Lipoprotein particles that contain only ApoA-1(LpA-1 particles) can increase cellular cholesterol efflux from cultured cells *in vitro*, whereas LpA-1:A-2 particles do not [17]. Several studies support the fact that LpA-1 is more effective than LpA1:A-2 in promoting cellular cholesterol efflux, the first step in reverse cholesterol transport, leading to inhibition of dietary or genetically induced atherosclerosis [18]. Moreover, among the HDL molecules exclusively containing Apo-A1, several subclasses have been separated by gel filtration chromatography. These have been denoted large, medium and small LpA-1 and contain different amounts of lipids (much higher in large Lp-A1). This determines the differences in their cholesterol-reducing capacity, being lower in large LpA1 compared with medium and small LpA1 [19]. This huge variety of circulating HDL and ApoA1 molecules explains the different isoforms of ApoA1 differentiated by 2DE in our different comparison sets.

Our study affords three important observations regarding Apo-A1 dynamics in young obese children. First, the observation of the negative correlation found between BMI-SDS and most Apo-A1 isoforms in the whole cohort (OB + C) indicates that this is one of the initial derangements in lipid metabolism and that it can already be present at a very young age. Secondly, when obese children display IR, Apo-A1 production is more severely impaired. Finally, it is important to emphasize that the sensitivity of the standard methodology used in daily practice to determine HDL and Apo-A1 is insufficient to detect these initial changes in lipid metabolism demonstrated by 2DE under the presence of IR. As previously stated, this limited sensitivity can be influenced by the large variety of Apo-A1 and HDL isoforms [19] that can be selectively modified and/or change in different directions. In contrast, serum proteomic analysis by 2 DE is able to differentiate particular Apo-A1 isoforms and to detect the subtle and specific changes induced by obesity associated IR in very young obese children. Hence, this methodology could complement commonly used techniques in this subgroup of patients (insulin resistant obese).

Another apolipoprotein (J), also called clusterin, SGP2, TRPM-2, or CLI, has shown great variability in response to different pathological conditions. This is a plasma protein with cytoprotective and complement-inhibiting activities that acts through a specific receptor (megalin) and is also part of the HDL molecules. HDL molecules that contain Apo J and Apo A-I carry paraoxonase (PON1) that protects low-density lipoproteins from oxidative modification [20]. Since its discovery in the 80s, it has been proposed to influence inflammation and autoimmunity and to be involved in several pathophysiological processes such as carcinogenesis, kidney injury, senescence or Alzheimer disease [21].

To our knowledge, no data are available in the literature regarding serum clusterin dynamics in childhood obesity. Although the study by Kujiraoka et al. postulates that serum clusterin levels are unrelated to gender, BMI or age, it is reported to be increased in coronary heart disease with a postulated anti-atherogenic effect and its production enhanced by stress and in T2DM adults [22]. However, the elevation of clusterin in diabetes has been challenged, as it disappeared after adjusting for the level of glycemia [20]. Clusterin is known to accumulate in the artery wall during the development of atherosclerosis [22] and has been localized in the infarcted heart during myocardial infarction [23]. Moreover, a protective effect of exogenous clusterin has been demonstrated on ischemically challenged cardiomiocytes *in vitro*[24].

The differences between groups in clusterin levels observed in this study lost their significance after FDR analysis. However, the recurrent observation of these differences in every subset of comparisons established and the negative correlations with BMI-SDS, HOMA index and visfatin levels of some of its isoforms suggest an eventual relevance of this protein in the initial phase of metabolic impairment in obese children, differing from those observed in adults, not increasing but decreasing. This could suggest the loss of its postulated anti-atherogenic and lipid-lowering effects in direct relationship to of the degree of excess BMI and being further intensified with the onset of IR. It is possible that there is a lack of a stress stimulus powerful enough to induce the increase in serum clusterin described in adults as at these early ages gross metabolic impairment or arteriosclerotic changes are absent. The commercially available ELISA assay was unable to detect the trends to change in clusterin observed by 2DE, again reinforcing the need for validation of the changes in clusterin levels observed by 2DE in stages of initial metabolic derangement.

The third group of molecules where multiples changes were found is the set of haptoglobin isoforms. Obesity tended to decrease haptoglobin isoforms of higher MW and increase low MW isoforms, with this last change found to be significant after FDR analysis. This change was enhanced when comparing obese IR children with obese non-IR children, whereas weight loss causes the opposite effect, inducing an increase in higher MW haptoglobin isoforms and a decrease in the low MW ones. This could explain the limited ability of ELISA assays, which detect the different isoforms of haptoglobin as a pool, to discriminate these complex changes (as observed in the comparison between IR and non-IR).

Apart from the loss of significance of the differences between groups of several isoforms of haptoglobin after FDR analysis, two arguments can be raised to support the eventual pathophysiological relevance of these observations. These arguments include the abundance of haptoglobin isoforms in the serum proteome and the possible influence of inter-individual genetic variability. In effect, the two alleles for haptoglobin (1 and 2) give rise to three major phenotypes (Hp1-1, Hp2-2 and Hp2-1), each of them with different capacities to form homo- or hetero-polymers of haptoglobin [25]. However, the paired comparison performed after weight loss in the same set of children, determining an increase in the higher MW isoforms and a decrease in the low MW forms, supports a possible pathophysiological importance of our observation. Further support comes from the observed correlations between the excess of BMI and haptoglobin isoforms, positive for those with low MW and negative for those with high MW, and between HOMA index and the low MW isoforms of haptoglobin.

The increase in the synthesis of low MW isoforms of haptoglobin in obesity, and further when it associates IR, could be interpreted as a component of the low-degree inflammation state accompanying obesity, which is already present in early ages [6]. Haptoglobin is an acute phase protein exerting antioxidant activity through several mechanisms, including activation of neutrophils, maintenance of reverse cholesterol transport and inhibition of cyclooxygenase and lipooxygenase [26]. This antioxidant function is more pronounced in individuals with the Hp1-1 phenotype. Interestingly, this phenotype generates the smallest haptoglobin molecules (dimers), compared with the Hp2-1 (trimers and tetramers) and Hp2-2 (trimers, tetramers and polymers) phenotypes, with this last phenotype showing the lowest antioxidant capacity [25,26]. In this regard, the positive correlation found between serum IL-6 levels and the expression of two of the low MW isoforms of haptoglobin (including spot #8102) in our obese cohort suggests the feasibility of the proposed explanation, especially taking into account that IL-6 induces haptoglobin synthesis [25,27].

The lack of significance after FDR analysis of the increase in alpha 1 antitrypsin levels, another acute phase protein, as a consequence of IR in 2DE analysis as in ELISA does not allow any conclusion to be made, although its variations might be expected due to the existence of a proinflammatory environment. The increase in alpha-1-acid glycoprotein detected by ELISA and suggested by 2DE (although not reaching statistical significance after FDR) has been previously reported in obese adults [28] and T2DM [29], though its biological significance remains uncertain.

Decreased serum levels of some isoforms of two binding proteins, transthyretin and vD-BP, were observed in obese children, although they lost their significance after adjusting by FDR. Transtyretin is bound to RBP4 in plasma and an elevation in RBP4 is thought to contribute to the development of IR associated with obesity and T2DM [30] A decrease in transthyretin could result in increased free RBP4 levels contributing to IR in obese subjects, with weight loss improving this impairment. Similarly, low vitamin D levels have been consistently reported in obese patients of all ages [31]. Although vitamin D liposolubility seems to be the main determinant of its low serum levels in obese subjects, the detected decreases in vitamin D binding protein isoforms could also be involved. Again 2DE results for trend to change in these two proteins need further validation, but could confer 2DE a potential role in early detection of the decrease in specific isoforms of transtyretin and vD-BP, before changes in total transthyretin, RBP4 or vD-BP are observed.

Regarding the changes observed in protein spot intensity in the three sets of comparison, losing their significance after FDR analysis, it should be pointed that, although correction for multiple comparisons is considered statistically recommendable to avoid rejecting the null hypothesis too readily, some author postulate that it should be avoided. This is explained on the basis that reducing the type I error for null associations increases the type II error for those associations that are not null; thus suggesting that a policy of not making adjustments for multiple comparisons could be preferable because it will lead to fewer errors of interpretation when the data are actual observations on nature [32].

## Conclusions

Our results suggest that changes in some isoforms of circulating peptides, mainly related to inflammation and lipid metabolism, accompany the early onset of obesity and IR in childhood. Furthermore, weight loss can ameliorate these modifications, at least partially. Some of these changes are undetectable by methods routinely used in the clinic under certain conditions, such as IR. Proteomic analysis, in particular, detection of isoforms of known proteins (ApoA1 and low MW haptoglobin) could be useful to complement them. In addition, this proteomic approach has allowed for the identification of several candidate proteins (including ApoJ/clustering, vitaminD binding protein, transthyretin) whose dynamics in childhood obesity and IR need further validation, but could result in novel biomarkers for the risk of development of obesity related metabolic complications.

## Abbreviations

2-DE: Two dimension electrophoresis; α-1-BAGP: Alpha-1-acid glycoprotein; α1-AT: Alpha-1-acid antitrypsin; Apo-A1: Apolipoprotein A1; BMI: Body mass index; C: Controls; D: Study time-point at diagnosis; FDR: False discovery rate analysis.; HDL: High density lipoprotein; HOMA: Homeostasis model assessment; HMW-adiponectin: High molecular weight adiponectin; IL-6: Interleukin 6; IR: Insulin resistance; LDL: Low density lipoprotein; MS: Mass spectrometry; MS-MS: Tandem mass spectrometry; OB: Obese; OGTT: Oral glucose tolerance test; Pros-OB: Prospectively followed obese cohort; RBP4: Retinol binding protein 4; SA: HMW to T adiponectin ratio; SDS: Standard deviation score; T2DM: Type 2 diabetes mellitus; T-adiponectin: Total adiponectin; TNF-α: Tumoral necrosis factor alpha; vD-BP: Vitamin D binding protein; VLDL: Very low density lipoprotein; WL: Study timepoint after weight loss.

## Competing interests

The authors declare lack of any financial or non-financial competing interests.

## Authors’ contributions

GAMM and LSS carried out the proteomic studies, participated in data interpretation and drafted the manuscript. VB and SO carried out the immunoassays. DB participated in the result interpretation. JA and JJK conceived of the study, and participated in its design and coordination and helped to draft the manuscript. All authors read and approved the final manuscript.
